# Ataxin1L Is a Regulator of HSC Function Highlighting the Utility of Cross-Tissue Comparisons for Gene Discovery

**DOI:** 10.1371/journal.pgen.1003359

**Published:** 2013-03-28

**Authors:** Juliette J. Kahle, George P. Souroullas, Peng Yu, Fabian Zohren, Yoontae Lee, Chad A. Shaw, Huda Y. Zoghbi, Margaret A. Goodell

**Affiliations:** 1 Department of Molecular and Human Genetics, Baylor College of Medicine, Houston, Texas, United States of America; 2 Stem Cells and Regenerative Medicine Center, Baylor College of Medicine, Houston, Texas, United States of America; 3 Howard Hughes Medical Institute, Baylor College of Medicine, Houston, Texas, United States of America; 4 Jan and Dan Duncan Neurological Research Institute, Texas Children's Hospital, Houston, Texas, United States of America; 5 Department of Pediatrics, Baylor College of Medicine, Houston, Texas, United States of America; 6 Department of Neuroscience, Baylor College of Medicine, Houston, Texas, United States of America; Cincinnati Children's Hospital Medical Center, United States of America

## Abstract

Hematopoietic stem cells (HSCs) are rare quiescent cells that continuously replenish the cellular components of the peripheral blood. Observing that the ataxia-associated gene *Ataxin-1-like* (*Atxn1L*) was highly expressed in HSCs, we examined its role in HSC function through *in vitro* and *in vivo* assays. Mice lacking Atxn1L had greater numbers of HSCs that regenerated the blood more quickly than their wild-type counterparts. Molecular analyses indicated *Atxn1L* null HSCs had gene expression changes that regulate a program consistent with their higher level of proliferation, suggesting that *Atxn1L* is a novel regulator of HSC quiescence. To determine if additional brain-associated genes were candidates for hematologic regulation, we examined genes encoding proteins from autism- and ataxia-associated protein–protein interaction networks for their representation in hematopoietic cell populations. The interactomes were found to be highly enriched for proteins encoded by genes specifically expressed in HSCs relative to their differentiated progeny. Our data suggest a heretofore unappreciated similarity between regulatory modules in the brain and HSCs, offering a new strategy for novel gene discovery in both systems.

## Author Summary

Our labs, working separately on brain function and blood stem cells, noticed that a particular gene involved in movement disorders was also expressed in the blood system. We discovered through bone marrow transplantation experiments that this gene, called Ataxin-1-like, normally plays a role in restricting the number of blood-forming stem cells; stem cells lacking this gene were more numerous and more active. We wondered if this brain-blood similarity would hold for a larger number of genes, so we used bioinformatics approaches to compare large datasets our labs had generated from each system. We found that a surprising number of genes implicated in autism and ataxia by molecular studies were also highly expressed in blood-forming stem cells. We suggest that such cross-system comparisons could be used more widely to discover genes with important functions in brain and blood, but also perhaps other systems.

## Introduction

Lifelong blood production is sustained by a quiescent reserve of hematopoietic stem cells (HSCs), which have the capacity to generate both additional stem cells (self-renewal) and differentiated blood cells. The balance between self-renewal and differentiation is tightly regulated and also flexible, ensuring adequate blood production under a variety of conditions while also maintaining a stem cell pool. While knock-out (KO) mice have allowed the identification of a number of genes that influence this balance, the relative scarcity of HSCs in the bone marrow limits the application of some genome-wide technologies that would uncover additional critical players and the basic biology of their regulation.

In contrast to the active turnover of the hematopoietic system, the brain is relatively static; it is primarily composed of terminally differentiated neurons and glia, but also contains rare self-renewing stem cells. We knew from the literature that a number of genes that exhibit roles in neurogenesis and neuronal function also play a key role in hematopoiesis. For example, *Gfi1* is critical for Purkinje cell function in the brain [1], as well as maintenance of hematopoietic stem cell function and myeloid development [2]. In addition, *Scl/Tal1* is critical for HSC development and function [3] and also for normal brain development [4].

With these examples in mind, when *Ataxin-1L*, which has been implicated to play a role in neurological disease [5], [6], but has no known hematopoietic function, was highly expressed in a microarray from HSCs [7], we wanted to test whether it too played a role in both tissues. We thus tested its function in the hematopoietic system through *in vitro* and *in vivo* assays using Atxn-1L null mice. We discovered that Atxn-1L is a strong negative regulator of hematopoietic stem cells, as knock-out mice exhibit greater numbers of more active stem cells. These data, together with the literature examples above, led us to examine the brain-blood relationship in a systematic way using bioinformatics strategies. Here, we show that genes and proteins identified functionally or by computational approaches as relevant in the brain are also implicated in hematopoiesis by multiple criteria, supporting the value of cross-tissue comparisons for gene discovery.

## Results

### *Atxn1L* KO mice display augmented HSC function

*Atxn1L* is a paralog of *ATXN1 (aka ATAXIN1)* [5], [8], originally identified in humans as the gene mutated in Spinocerebellar ataxia type 1 (SCA1) [9], [10]. *ATXN1* has a triplet repeat sequence that becomes expanded and pathogenic in SCA1 patients, resulting in progressive ataxia with age. *Atxn1L* expression is highly overlapping with that of *Ataxin1*, and the two genes are at least partially functionally redundant [5]. Double Atxn1/Atxn1L knockout (KO) mice have a set of severe phenotypes absent in either of the single KO mice and die shortly after birth [11]. The phenotypes of the double KO mice include hydrocephalus, omphalocele, and a lung alveolarization defect. Hematopoietic system phenotypes of the single or double KO mice have not been previously reported.

Here we focused on *Atxn1L* because of its high expression in the hematopoietic system. Having a mouse with a null allele for Ataxn1L in our lab [11] and little prior information about any potential function for this gene in the hematopoietic system, we proceeded to study its function in HSCs.

**Figure 1:**
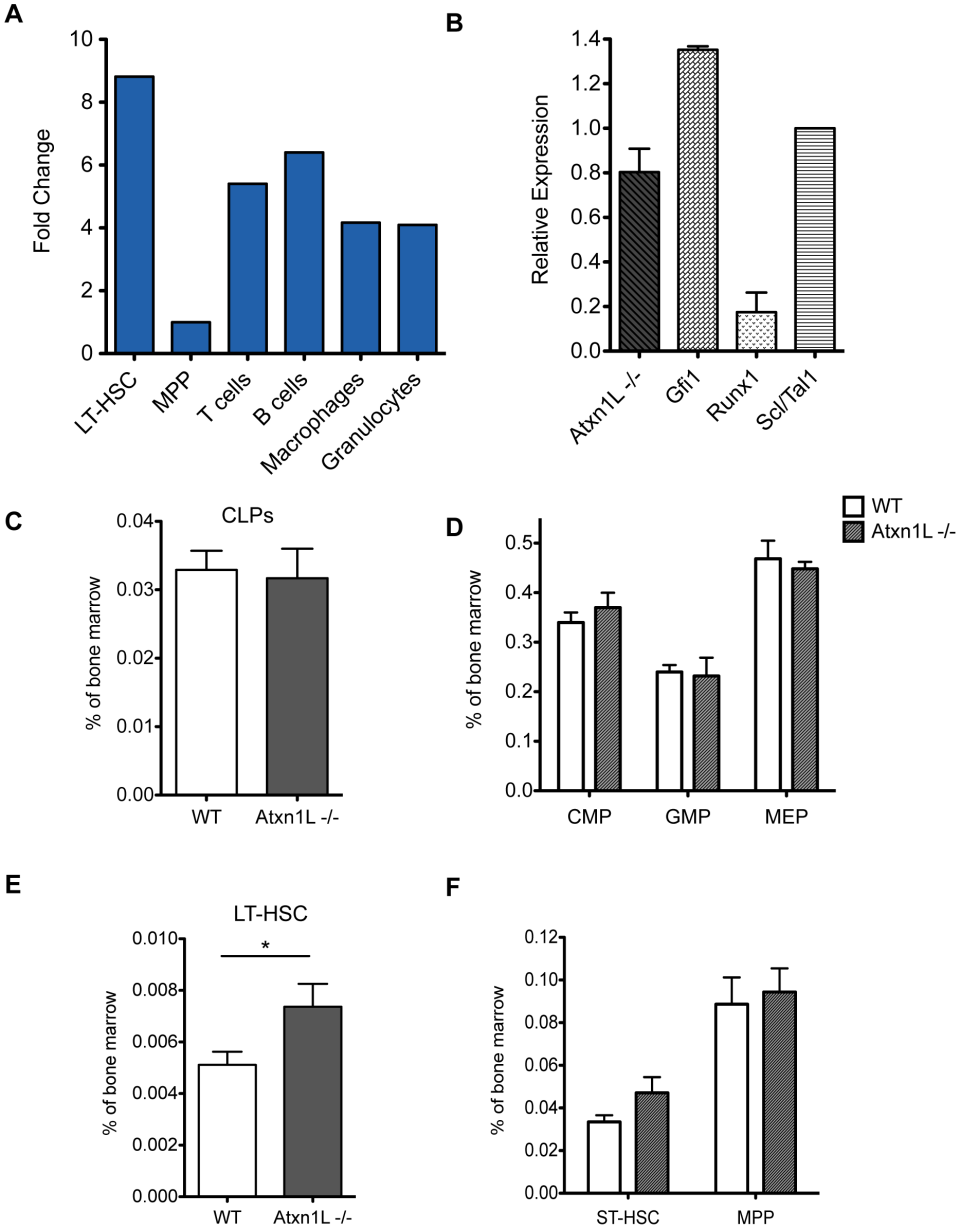
Expression of *Atxn1L* in the hematopoietic system and frequency of progenitors in knock-out animals. A. Real-time rt-PCR analysis of *Atxn1L* expression in the indicated purified populations, normalized to the level in MPPs. LT-HSC: long-term hematopoietic stem cells; MPP: multipotent progenitors (MPP). Terminally differentiated cells were purified from the peripheral blood. B. Real-time rt-PCR analysis of expression of the indicated genes in purified LT-HSCs, normalized to *Scl/Tal1*. Data are representative of two independent experiments. C, D. Analysis of the proportion of the indicated populations in the bone marrow of WT vs *Atxn1L^−/−^* mice. CLP: common lymphoid progenitors, CMP: common myeloid progenitors, GMP: common myeloid progenitors, MEP: megakaryocyte-erythroid progenitors. E. Analysis of the proportion of LT-HSCs cells in the bone marrow of WT vs. *Atxn1L^−/−^* mice (t-test, *P = 0.047*). F, Analysis of the proportion of ST-HSCs and MPPs. ST-HSC: short-term HSCs. LT-HSCs: Lineage^−^, Sca-1^+^, c-kit^+^, CD34^−^, Flt3^−^; other populations defined as in methods. In C–F, n≥5; bars indicate the mean plus standard error.

*Atxn1L* is expressed in multiple hematopoietic lineages, but most highly in the stem cells, with an expression level comparable to that of other key hematopoietic regulators such as *Gfi1* and *Scl/Tal1* (Figure 1). To determine whether Atxn1L plays a role in HSC function, we first examined complete blood counts of adult *Atxn1L^−/−^* mice and the proportions of myeloid and lymphoid cells in the peripheral blood. There were no significant differences from the numbers in their wild-type counterparts (data not shown). Similarly, bone marrow progenitor populations were present at normal frequency, however, there was a slight increase in the proportion of long-term HSCs (*P* = 0.047) (Figure 1).

**Figure 2:**
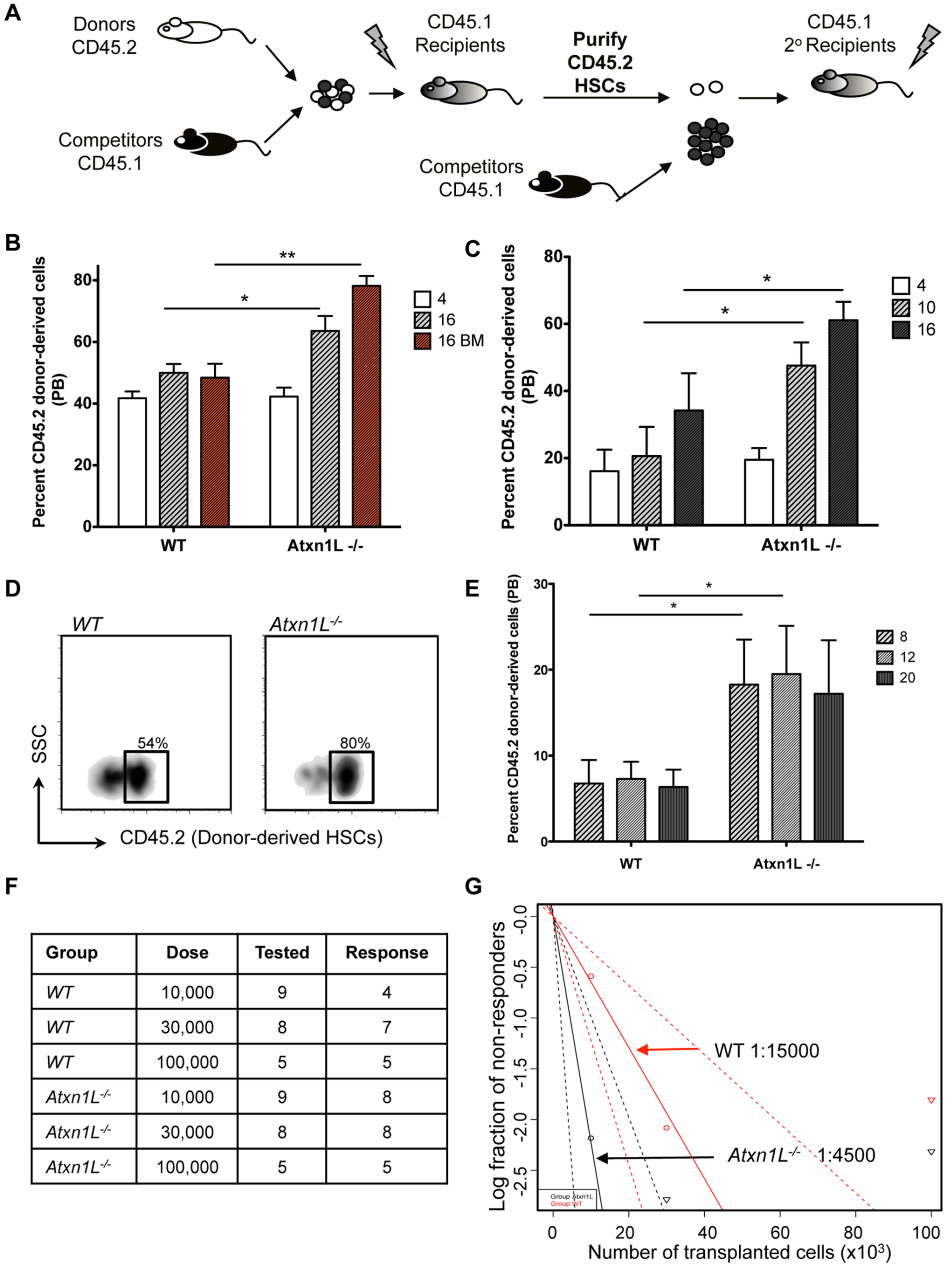
*Atxn1L^−/−^* mice have enhanced HSC function. A. Schematic of the experimental design. Equal numbers of bone marrow (BM) cells from WT (competitors) and *Atxn1L* ^−/−^ or WT (donor) cells were transplanted into lethally irradiated recipients. For secondary transplants, HSCs were purified from primary recipients and transplanted into new recipients along with fresh whole BM competitor cells. B. Competitive whole BM transplants comparing the engraftment ability of WT vs *Atxn1L^−/−^* cells. White and grey bars indicate peripheral blood contribution at 4 and 16 weeks post transplant. Red bars indicate donor cell contribution to bone marrow after 16 weeks. C. Peripheral blood chimerism after purified HSC transplantation at the indicated weeks. Twenty purified HSCs from WT and *Atxn1L^−/−^*mice were transplanted along with 250,000 WT competitor BM cells. D. Analysis of the proportion of donor-derived HSCs obtained from transplant recipients as pooled from 5 mice. HSCs were defined as SP^KSL^+CD150+ cells. E. Peripheral blood chimerism after secondary transplants from HSC-transplanted mice from (D) at the indicated weeks (n = 5). F. Limiting dilution competitive repopulation assay with the indicated numbers of WT and *Atxn1L^−/−^* BM cells. The table shows the number of mice tested in each group and the number of mice that were engrafted with donor cells (contribution to blood>0.1%). G. The graph shows the percentage of mice that contain less than 0.1% multi-lineage engraftment 12 weeks post transplant. The HSC frequency was calculated using the L-Calc software according to Poisson statistics (two-tailed t-test; p = 0.016). (* *P<0.05*, ** *P<0.01*). All bone marrow transplantation experiments were repeated at least twice with similar results. All graphs display the mean plus standard error.

Because *Atxn1L* is most highly expressed in HSCs, we next examined HSC function via bone marrow transplantation studies. We first carried out competitive whole bone marrow transplantation assays in which KO bone marrow was competed against WT bone marrow from syngeneic strains of mice that are distinguishable using the CD45.1 and CD45.2 allelic system (Figure 2A). Equal numbers (250×10^3^) of *Atxn1L^−/−^* donor and WT competitor whole BM cells were transplanted into lethally irradiated recipients and their contribution to peripheral blood production was assessed at 4-week intervals. The contribution of *Atxn1L^−/−^* BM to peripheral blood regeneration four weeks after the transplant was equivalent to WT; however, over time, the contribution of *Atxn1L^−/−^* increased significantly such that 70–80% of the blood was derived from the bone marrow of *Atxn1L* KO mice, whereas the controls, in which WT bone marrow was competed with WT bone marrow, remained around 50% (Figure 2B). The difference in repopulation activity between WT and KO bone marrow at 16 weeks, when the majority of the blood cells are considered derived from stem cells within the transplanted bone marrow was highly significant (*P<0.01*).

Because the bone marrow of *Atxn1L* KO mice harbored a slightly higher proportion of phenotypically-defined HSCs (Figure 1), we wanted to determine whether the higher peripheral blood reconstitution activity of *Atxn1L^−/−^* bone marrow was simply due to a higher number of HSCs, or a higher inherent repopulating activity of mutant HSCs. To test this, we examined the repopulation ability of HSCs purified from KO and WT bone marrow. Twenty HSCs (side population (SP), c-Kit^+^ Sca1^+^ Lineage^−^ (KLS) CD150+) were mixed with 250,000 WT whole bone marrow cells and transplanted into lethally irradiated recipient mice. We found that *Atxn1L^−/−^* HSCs were superior in regeneration of the hematopoietic system, providing nearly double the blood contribution at 16 weeks after transplant (*P*<0.05) (Figure 2C). When the bone marrow of these recipient mice was examined 16 weeks after transplantation, almost 80% of the HSCs were donor-derived in mice transplanted with *Atxn1L* null HSCs, compared to 50% in the WT control group (*P*<0.05) (Figure 2D). This significantly better reconstituting activity of purified *Atxn1L^−/−^* HSCs indicates that *Atxn1L^−/−^* null mice harbor more HSCs that are inherently more active.

Bone marrow or stem cell transplantation assesses the ability of HSCs to repopulate the bone marrow and differentiate, but the ability of stem cells to self-renew is most rigorously assessed by secondary transplantation. To examine whether the self-renewal capacity of *Atxn1L^−/−^* HSCs was also enhanced, we re-isolated HSCs from the bone marrow of primary transplant recipients and re-transplanted them with fresh competitor bone marrow into secondary recipients (Figure 2A). Again, the performance of the *Atxn1L^−/−^* HSCs was superior to WT HSCs (*P*<0.05), with nearly double the activity in this rigorous assay (Figure 2E).

Finally, since both primary and secondary HSC transplants rely on the phenotypic definition of HSCs, we carried out a limiting dilution assay to assess the presence of functional repopulating units in WT compared with *Atxn1L^−/−^* bone marrow. This assay does not rely on competition, and is considered the most exacting to observe relative functional HSC activity [12]. In mice transplanted with the lowest doses of WT bone marrow (10,000 cells), less than half of the mice were engrafted. In contrast, 8/9 animals transplanted with the same amount of bone marrow from KO mice were engrafted (Figure 2F, 2G). These data indicate that the frequency of repopulating units in *Atxn1L^−/−^* marrow is ∼1/4500 cells, while the frequency in WT marrow is ∼1/15,000 cells (the latter being in line with standard estimates).

Together, these data establish that *Atxn1L^−/−^* mice have enhanced HSC activity. The slightly higher frequencies of phenotypically defined HSCs in bone marrow can not account for the significantly higher functional HSC activity observed by bone marrow and stem cell transplantation experiments. Thus, Atxn1L appears to be a negative regulator of HSC function.

### *Atxn1L* does not influence homing of HSCs to the recipient bone marrow

**Figure 3:**
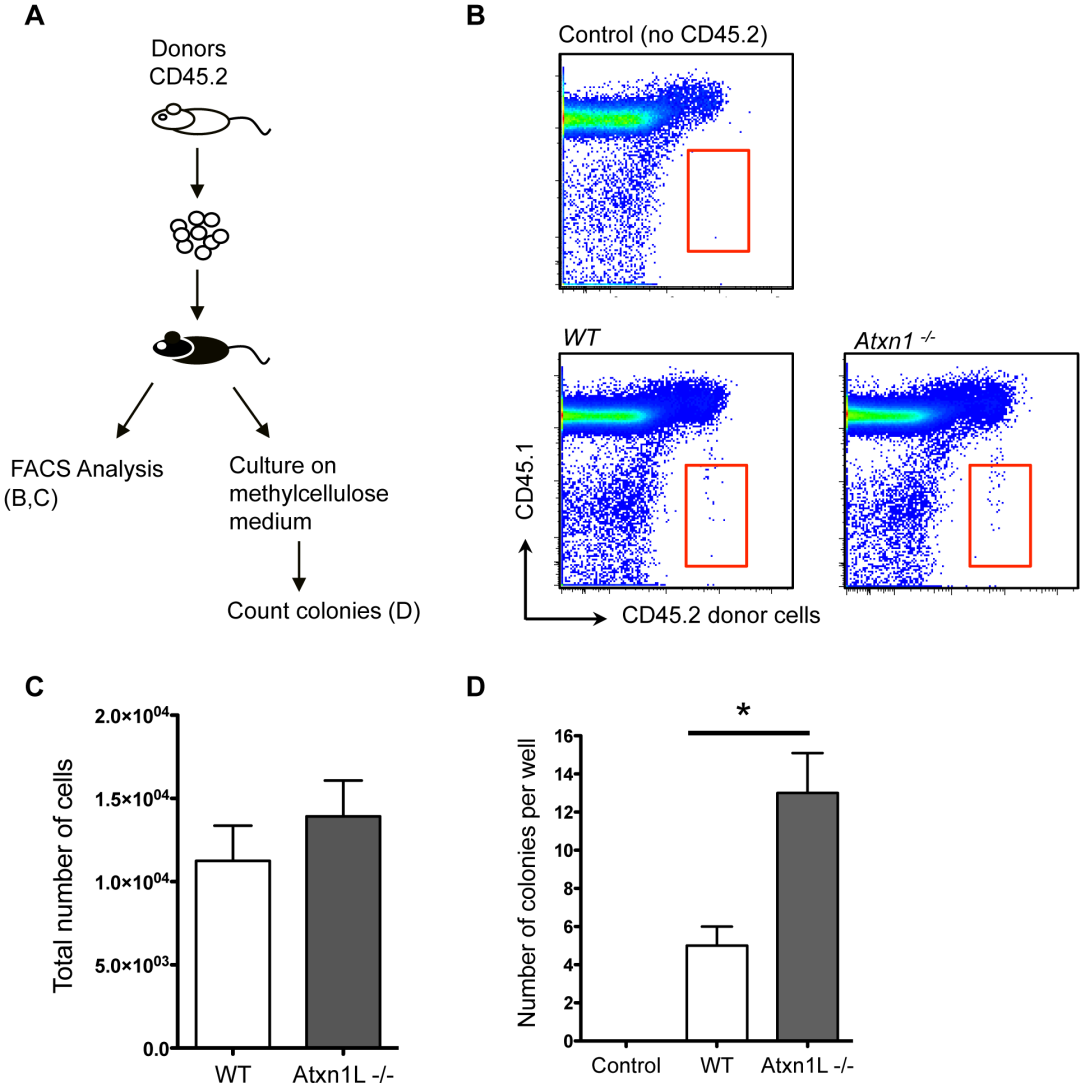
Loss of *Atxn1L* does not affect the ability of HSCs to home to the recipient marrow after transplantation. A. Schematic of experimental design. 30,000 CD45.2 hematopoietic progenitors (lin−, Sca-1+, c-kit+, [KSL]) were purified and transplanted into CD45.1 recipient mice (n = 7 per genotype). (B) 18 hours post transplant, bone marrow from the recipient mice was analyzed by flow cytometry for the presence of CD45.2 donor-derived cells. Representative of two independent experiments. C. The graph shows the absolute number of WT vs Atxn1L^−/−^ donor-derived cells in the BM of recipient mice. (Not significantly different.) D. A portion of the KSL cells that were transplanted into CD45.1 recipient mice was also plated on methylcellulose medium to assess cell proliferation. The graph shows the average number of colonies from triplicate plates derived from WT vs. Atxn1L^−/−^ progenitor cells after 12 days in culture (*P<0.01*). Error bars indicate standard error of the mean.

One known factor that contributes to repopulation efficiency after bone marrow transplantation is the ability of the HSCs and their progenitors to home to the bone marrow niche. Thus, we tested whether the superior activity of *Atxn1L^−/−^* HSCs could be attributed to enhanced bone marrow homing. We intravenously transplanted 30,000 purified hematopoietic progenitors (c-Kit+, Sca-1+ Lineage− (KSL) cells into lethally irradiated recipient mice, and sacrificed the recipients 18 hours later to examine the proportion of progenitors reaching the femurs and tibias using flow cytometry (Figure 3A). We found no difference between the number of WT and *Atxn1L^−/−^*cells that were able to home within 18 hours (Figure 3B, 3C). In addition, we plated a portion of the bone marrow extracted from these recipients into methylcellulose media to assess colony formation activity from donor cells, a further indication of homing efficiency. In contrast to the direct homing assay, we found a significant increase (*P*<0.01) in the number of colonies generated from the *Atxn1L^−/−^* donor cells compared to WT (Figure 3D). This finding suggests that although similar numbers of progenitors are reaching the bone marrow, the *Atxn1L^−/−^* progenitors are significantly more proliferative. Since long-term HSCs are only a small portion (∼10%) of the KSL fraction, the above result largely reflects the properties of short-term HSCs and committed progenitors. Together, these data indicate that increased homing of hematopoietic progenitors is not likely to be a major factor contributing to the enhanced repopulating potential of the *Atxn1L^−/−^* HSCs, and underscores the observation of augmented activity from the mutant HSCs.

### Loss of *Atxn1L* enhances HSC proliferation both *in vitro* and *in vivo*

**Figure 4:**
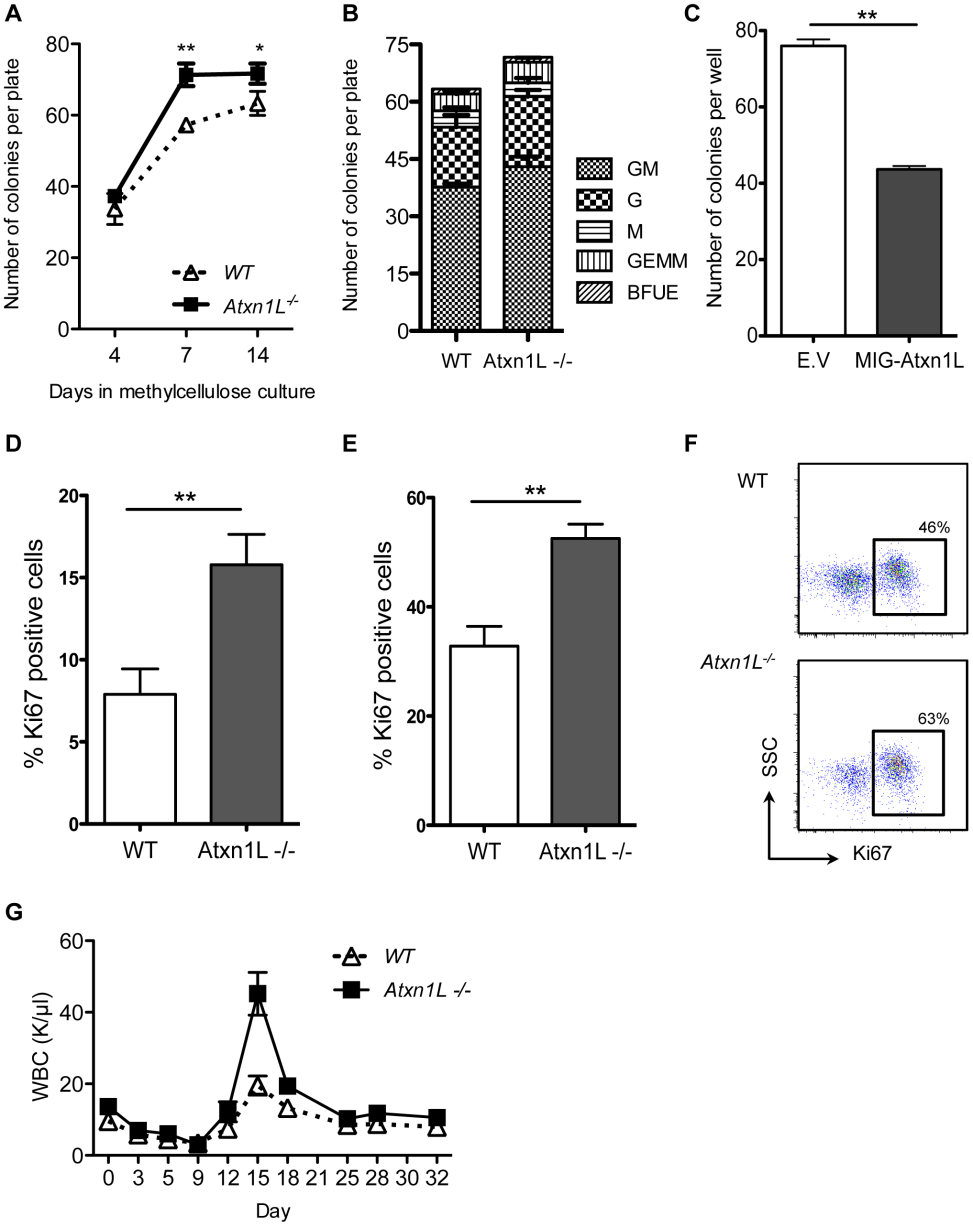
Loss of *Atxn1L* results in more proliferative hematopoietic stem and progenitor cells. A. Individual HSCs were sorted into 96-well plates containing methylcellulose media and colonies were counted and scored based on their morphology at the indicated time points. B. Proportions of colony types. C. Colony numbers from BM cells transduced with *Atxn1L*-overexpressing retrovirus compared to a GFP-only control vector (E.V). Results represent the average of three 96-well plates (*P<0.01*). D and E. *In vivo* proliferation analysis of WT vs *Atxn1L^−/−^* HSCs (D) (KSL, Flk2^−^, CD34^−^) and hematopoietic progenitors (E) (KSL) by Ki67 staining (n = 5, *P<0.05*). F. Representative flow cytometry plots of Ki67 staining on hematopoietic progenitors (KSL). G. Complete blood counts over time of blood from WT and *Atxn1L^−/−^* mice after a single injection of 5-FU. (n = 10/genotype, *P = 0.0007*). The graph shows representative data from three independent experiments. All graphs display the mean plus standard error.

To test the hypothesis that *Atxn1L^−/−^* HSCs are more proliferative, we performed both *in vivo* and *in vitro* assays. We sorted single HSCs from unperturbed *Atxn1L^−/−^* mice into hematopoietic colony-promoting methylcellulose media in 96-well plates, counted the total number of colonies at multiple times points, and analyzed colony morphology to determine their myeloid differentiation potential. We found a higher number of colonies derived from the *Atxn1L^−/−^* vs. WT HSCs when counted after 7 days (*P*<0.01), although this difference was not as significant after 14 days, suggesting that the rate of growth of the colonies was faster in the *Atxn1L^−/−^* group than the WT, consistent with a higher proliferation rate. There was no difference in the colony types produced (Figure 4B).

To determine whether overexpression of Atxn1L would cause the opposite phenotype, we cloned *Atxn1L* into a retroviral vector that also expresses GFP downstream of an IRES that is cloned in tandem with the Atxn1L coding sequence. Hematopoietic progenitor cells were transduced with the virus, cultured for 24 hours, and GFP+ cells were sorted into wells containing methylcellulose medium. Stem and progenitor cells over-expressing Atxn1L generated fewer colonies compared to cells transduced with an empty vector control (*P*<0.01) (Figure 4C), consistent with a role for Atxn1L in negatively regulating HSC proliferation.

To examine whether *Atxn1L^−/−^* cells exhibited altered proliferative activity directly, we purified HSCs and immunostained them for Ki67, a marker of cycling cells. As expected, only a small portion of WT HSCs were Ki67-positive, whereas the proportion of Ki67-positive cells in *Atxn1L^−/−^* KO HSCs was significantly higher (*P*<0.01) (Figure 4D). This was also true in a less purified progenitor population (c-kit+, Sca-1+, lineage− (KSL), *P<0.01*) (Figure 4E, 4F).

If *Atxn1L^−/−^* HSCs have enhanced proliferation *in vivo*, this may be manifested in a differential response to agents that are toxic to proliferating cells. We thus tested the response of WT and *Atxn1L^−/−^* mice to a single injection of 5-FU by measuring their CBCs over an extended period of time. Overall recovery time was unchanged; however, recovery of white blood cells reached a higher peak at days 15–17 (*P = 0.0007*) (Figure 4G), consistent with a greater proliferative response from mutant stem and progenitor cells (Figure 4D–4F). Recovery of platelets and RBCs showed no significant differences (not shown). To exclude the possibility that the phenotype is accounted for by greater resistance to apoptosis we analyzed stem and progenitor cells in WT and *Atxn1L^−/−^* mice, before and after 5-FU treatment, but there was no significant difference in the number of apoptotic cells (data not shown). While one might expect a higher stem cell cycling rate to be accompanied by slower HSC recovery time because 5FU would also kill the cycling KO HSCs, the faster recovery suggests the KO HSCs can become quickly activated. Furthermore, because the progenitors are also more in cycle, their rapid expansion may compensate for the effect of 5FU on the HSCs leading to faster recovery.

### Enhanced expression of genes involved in HSC proliferation in *Atxn1L^−/−^* HSCs

**Figure 5:**
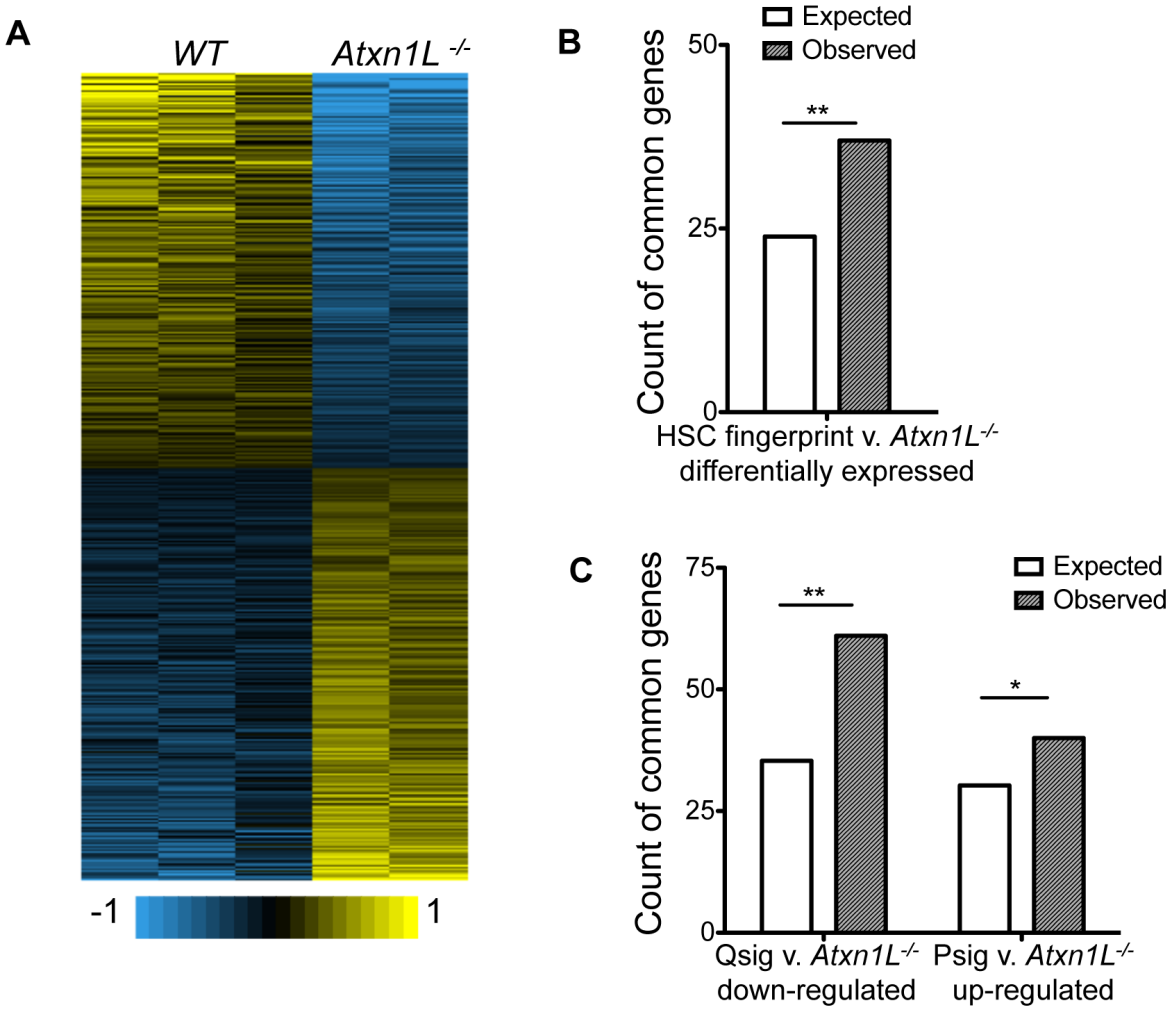
*Atxn1L^−/−^* HSCs are enriched for expression of HSC-specific genes and depleted for quiescence-associated genes. A. Heat map showing the expression profiles for each biological replicate from the microarray. B. Count of the expected and observed number of genes overlapping between the published HSC fingerprint genes [7] and genes that are differentially expressed between WT and *Atxn1L^−/−^* HSCs. (*P = 0.004152*) C. Count of the expected and observed number of genes overlapping between the published Quiescence-signature genes and genes that are down-regulated in *Atxn1L^−/−^* HSCs compared to WT (**, *P = 2.044×10^−5^*) and of the genes overlapping between the published Proliferation-signature genes and those that are up-regulated in *Atxn1L^−/−^* HSCs compared to WT. (*, *P = 0.06645*). HSCs were purified from 8-week-old mice and pooled for each chip.

*Atxn1L^−/−^* HSCs are more proliferative than WT HSCs and engraft better, but do not appear to cause leukemia, at least within the time frame of our analysis. To begin to gain insight into possible regulatory mechanisms that contribute to their high proliferative capacity, we determined the gene expression differences in *Atxn1L^−/−^* vs WT HSCs using expression microarrays (Figure 5). Consistent with the phenotype, where the KO HSCs are relatively normal in terms of their differentiation capacity but show an improvement of HSC activity, the gene expression differences were relatively modest. We found a total of 1013 genes that were different (484 up and 529 down; *P*-value≤0.05 and fold-change ≥1.5). Because the *Atxn1L^−/−^* HSCs resemble super-HSCs, we considered the possibility that their expression profile would reflect enhanced expression of HSC-critical genes. To test this, we compared the gene expression differences to the list of HSC-specific “fingerprint” genes [7]. We found a significant overlap of genes that are differentially expressed in *Atxn1L^−/−^* mice compared to WT and genes that are HSC fingerprint genes (Odds Ratio = 1.685, 95% CI 1.14–2.43, *P = 0.00598*). Some of the genes down-regulated in the KO are those identified functionally in other studies to have a key role in HSC maintenance and/or quiescence. For example both *HoxA7* and *HoxA9* are lower in the KO, and their loss has been associated with increased HSC proliferation [13], [14]. Similarly, *Pbx1* is lower in the KO, and also has been linked to maintaining HSC quiescence [15]. Some of the genes upregulated in the KO stem cells are associated with high stem cell quality. For example, *Tgf-beta-induced* (*Tgfbi*) is upregulated. TGFb signaling is associated with the most long-term HSCs, which have the greatest self-renewal capacity [16]. Similarly, upregulated in the KO HSCs, is *Plagl1*, which is a member of a group of imprinted genes that are associated with somatic stem cells of multiple tissues [17].

To investigate the link between *Atxn1L* and HSC proliferation, we compared the transcriptional changes in *Atxn1L^−/−^* HSCs with signatures of HSCs during proliferation vs quiescence [18]. We found that there is significant overlap between the genes that are down-regulated in *Atxn1L^−/−^* HSCs and the quiescence signature (Qsig) genes (Odds Ratio = 1.914, 95% CI 1.42–2.54, *P = 2.044×10^−5^*) and a nearly-significant overlap between the up-regulated genes with the proliferation signature (Psig) gene sets (Odds ratio = 1.376, 95% CI 0.96–1.93, *P = 0.066*) (Figure 5C). These data are consistent with an impact of loss of Atxn1L on maintenance of quiescence, as shown in Figure 2 and Figure 4. Aside from a few genes, we did not see differences at the pathway level in TGFβ, Wnt or Pten/AKT pathways. The finding that the gene expression changes in *Atxn1L* null HSCs are concordant with decreased quiescence and increased HSC proliferation provides insight into the potential molecules mediating the HSC phenotypes, although the precise molecular event(s) leading to these phenotypes remains elusive.

### HSC “fingerprint” genes are unexpectedly enriched in neuronal gene sets

**Figure 6:**
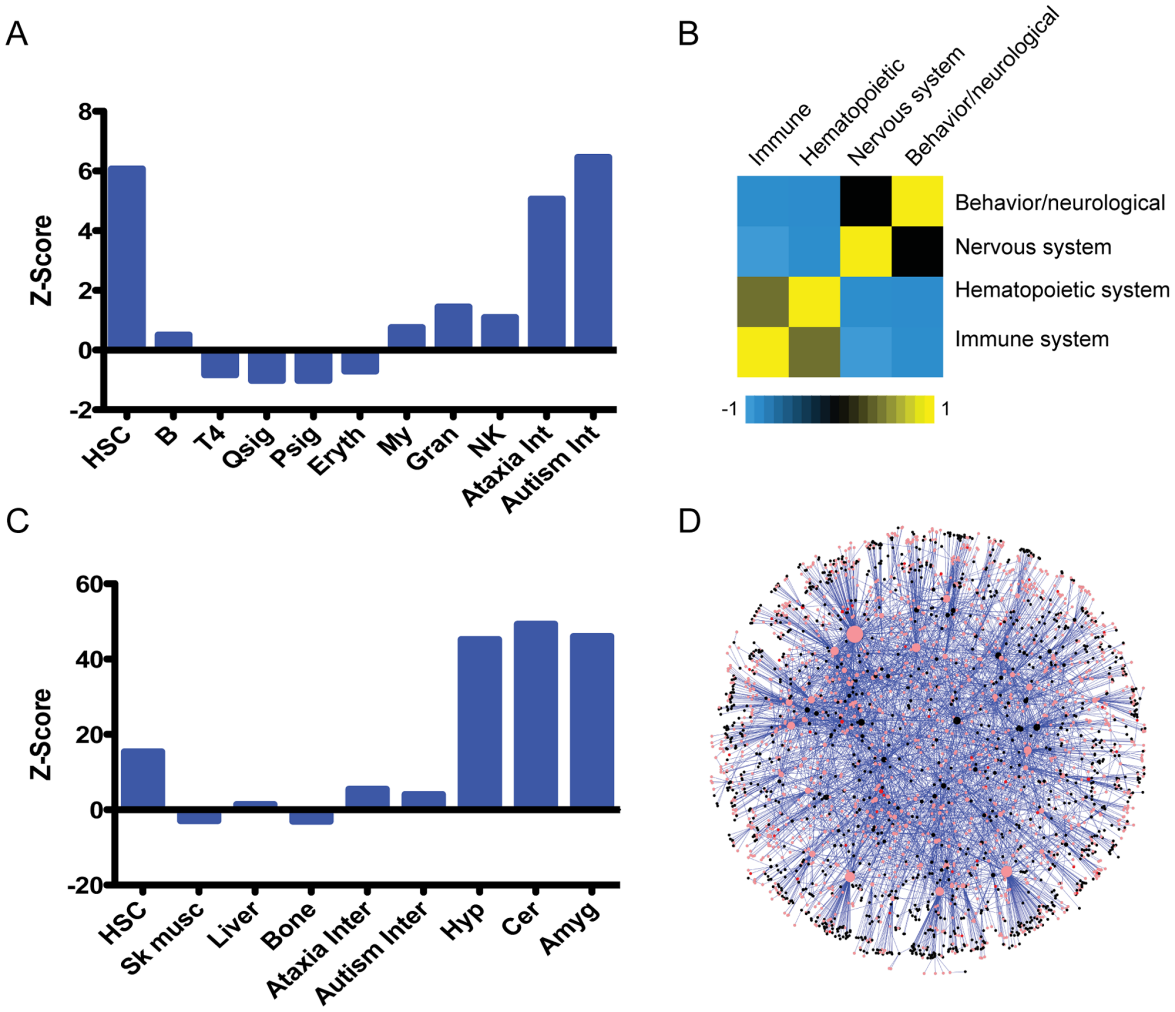
An unexpected relationship between the hematopoietic and nervous systems. A. Enrichment scores (a standardized measure of deviation between the observed overlap and expected overlap of two sets: ((observed – expected)/StDev(observed)), see methods for details) for the indicated gene sets compared to genes reported to have behavioral/neurological or nervous system phenotypes in the Mouse Genome Informatics (MGI) database; Fisher's exact tests were used to determine p-values. B. Analysis of the correlation between reported phenotypes across all genes from the MGI database, with −1 (blue) representing negative correlation, and 1 (yellow) representing high correlation. C. Enrichment analysis (same as in A above) for genes expressed in HSCs compared to the indicated gene sets. D. HSC expressed genes were mapped to the ataxia interactome. This subnetwork consists of HSC expressed nodes and their connected interacting partners that form the ataxia interactome. HSC expressed proteins are pink, HSC fingerprint proteins are red and black nodes are interacting partners. The node size corresponds to its connectedness with the rest of the network.

To determine whether additional brain-associated genes could be used to identify other hematopoietic regulators, we systematically examined genome-wide data sets available in our laboratories and in public repositories in order to test for correlations between genes important in the brain and hematopoietic system. We previously identified a set of genes uniquely expressed in HSCs relative to their differentiated counterparts (HSC “fingerprint” genes) [7], as well as genes expressed in differentiated hematopoietic lineages but excluded from HSCs. Similarly, we have developed brain protein-protein interaction networks for several proteins that are abundant in the brain and are known to cause either ataxia or autism [19]–[21]. We used the genes from these data sets to probe the Mouse Genome Informatics (MGI) repository of knock-out mouse phenotypes in order to link genes within these datasets with neurological phenotypes in existing mutant mice (Figure 6A). As expected, the genes from proteins in the ataxia and autism interactomes are enriched for genes annotated to nervous system and behavioral phenotypes by the MGI. In other words, a significant number of those genes have already been reported to result in nervous system or behavioral phenotypes after genetic manipulation (most frequently knock-out). Surprisingly, the HSC fingerprint set (319 genes) was also highly enriched for genes reported to cause a nervous system or behavioral phenotypes after genetic manipulation (Fisher's Test *P = 3.39×10^−9^*), with a Z-score similar to the enrichment of genes derived from the autism interactome (Table S1). In contrast, lists containing genes specifically expressed in multiple other hematopoietic cell types, including multiple lymphoid and myeloid cell types did not show a similar enrichment for phenotypic annotations to nervous system/behavior phenotypes. The proliferation rate of normal HSCs is low, as for most cells in the brain. To determine whether enrichment for genes reflecting the proliferation state could account for the parallels between the HSC-specific genes and the nervous system/behavior phenotypes, we performed parallel analysis with a list of genes derived from quiescent vs proliferating progenitors [18]. However, these did not show enrichment with nervous system/behavior phenotypes, indicating that the enrichment in the HSC-specific gene list is not due solely to their proliferative state.

To examine this relationship using an independent list of genes, we took a compendium of genes shown to have an impact on HSC function when ablated in mice [22]. Among genes with a reported HSC phenotype, we found a significant enrichment in those likely to have a nervous system/behavioral phenotype (Z = 4.06; p = 5.72×10^−5^; Table S2).

### Blood versus neurological phenotypes

To ensure that this relationship between HSC-specific genes and the likelihood of a reported nervous-system-related phenotype was not simply due to a testing artifact based on properties of the MGI data, we examined the MGI database more systematically. We examined reported phenotypes for all genes in the MGI repository to determine the inherent correlation between genes tested for, and reported to have, KO phenotypes associated with the hematopoietic system, the immune system, the nervous system, and neurological/behavioral defects. In other words, we examined overlap, or similarity, between these phenotypic categories, determining the frequency with which a gene KO is reported to have both hematopoietic system defects as well as neurological phenotypes. As expected, genes that were reported to have an immune system phenotype were likely to also be reported as having a hematopoietic phenotype. Similarly, genes that were annotated as resulting in a nervous system phenotype after KO were likely to also have a behavioral phenotype (Figure 6B). However, there was very low overlap in genes listed as having a neuronal phenotype with those having a hematologic phenotype. These classes are distinctly lacking in commonly annotated genes. This contrast may arise because investigators often focus on one tissue, and therefore phenotypes in other tissues may be missed or under-reported. Nevertheless, these findings stand in sharp contrast to the significant overlap we empirically observed between our experimentally-derived HSC-specific gene sets and genes reported in MGI to have neurological phenotypes (Figure 6A). This strongly supports the concept of a unique relationship between the HSC-specific genes and the nervous system, and argues for the need of more cross-system phenotyping, and comprehensive reporting of phenotypes.

### Blood versus neurological gene expression and molecular interactions

We therefore hypothesized that we may find direct enrichment of genes expressed in HSCs in the neuronal disease protein interactomes as opposed to the indirect analyses through the MGI phenotypes. To examine this, we tested whether genes expressed in HSCs were enriched in the ataxia and autism interactome, and in gene expression data from various brain regions including the hypothalamus, cerebellum and amygdala in wild-type mice [23]–[25]. We found that HSC-expressed genes were highly enriched in all brain regions as well as in gene sets encoding proteins in the ataxia and autism interactomes (Figure 6C; Table S1). To determine the specificity of the relationship to the brain, we also compared the HSC-expressed genes to those found in data sets from skeletal muscle, bone, and liver. There was no significant enrichment in these tissues, indicating that the HSC-brain relationship is relatively unique.

The ataxia protein interactome was then independently used to generate a subnetwork comprised of HSC-expressed genes and their interacting partners (Figure 6D) and we plotted the protein interactions of this sub-network based on evidence from the ataxia interactome. The dense interconnected network that emerges suggests that at least some of the protein-protein interactions observed in the brain are likely to also take place in the HSCs based on their gene expression. The overlap with the interactome data suggests that the relationship between HSC and neuronal genes appears to be rooted in molecular interactions, and is not simply a feature of the similarity of the phenotypes reported for the relevant genes.

Finally, we examined the identity of the genes that overlap in the narrowly defined data sets. When HSC-specific genes (274 that are mapped to the human genome) are overlapped with genes encoding proteins in the Autism interactome (2437 human genes), we identify 36 genes (Table S3). Similarly, the overlap between HSC fingerprint genes and those from the Ataxia interactome (3436 genes) is 45 genes (Table S4). The genes included in both interactomes that are also HSC-specific number only 17, of which three are known to have a function in the hematopoietic system (e.g. *Gata2*). Several are implicated in the neurologic system (e.g. *Col4a2*), but few have a precisely-defined role (Table 1). Of the remainder, most have functions described outside the hematopoietic and nervous systems (e. g. *Tle1*, a Groucho-like repressor). Intriguingly, three of these (*Grb10*, *Peg3*, and *Ndn*) are monoallelically expressed depending on their parent-of-origin (imprinted), a gene group known to be enriched in multiple types of somatic stem cells [17]. Three others are members of the Wnt signaling pathway (*Mdfi*, *Tcf7l1*, *Enah*). All of these 17 genes would be intriguing candidates for further study in the hematopoietic or nervous systems.

**Table 1:** Genes present in the autism interactome, the ataxia interactome, and the HSC fingerprint.

Symbol	Name	Brain role	HSC role	Pathway or function
**Ndn**	Necdin	Hypothalamic neuron control	HSC activation	TF; imprinted gene; Prader-Willi region; interacts with nerv growth factor receptor
**Tac1**	Tachykinin 1	Hormonal neuro transmitter; critical for hippocampal fucntion	Unk	Peptide generates substance P and neurokinin peptides; KO results in lower pain response
**Ltbp3**	Latent transforming growth factor beta binding protein 3	Unk	Unk	Tgfb-binding protein; extracellular matrix; KO with craniofacial defects
**Col4a2**	Collagen type 4 alpha2	mutations cause porenchepahlay	Unk	Collagen; in all basement membranes
**Enah**	enabled homolog (Drosophila)	Unk	Unk	Homolog of Drosophila Enabled gene; cancer association; Wnt pathway target
**Grb10**	Growth factor receptor bound protein 10		Unk	Potent growth inhibitor; receptor adaptor protein; imprinted
**Mdfi**	MyoD family inhibitor	Unk	Unk	MyoD family inhibitor; negative regulator of Wnt signaling
**Jag2**	Jagged 2	Unk	HSC regulator	Ligand for Notch receptors; mutations cause craniofacial defects and lethality
**Gata2**	GATA binding protein 2	May control GABAergic neuronal cell fate	Critical HSC regulator	Transcription factor essential for blood development
**Peg3**	Paternally expressed gene 3	mutation disrupts oxytocin production	Unk	Transcription factor; imprinted
**Tle1**	Transducing like enhancer of split1	Unk	Unk	Homolog of Drosophila Enhancer-of-split; Groucho-related protein; Wnt signaling
**Pgr**	Progesterone receptor	Unk	Unk	Progesterone receptor
**Scmh1**	sex comb on midleg homolog 1	Unk	Unk	Homolog of Drosophila sex-combs on midleg; polycomb complex;
**Krt18**	Keratin 18	Unk	Unk	Keratin 18; intermediate fillament protein
**Maged1**	Melanoma antigen family D1	Unk	Unk	Melanoma antigen; Interacts with neurortrophin receptor; X-chromosome
**Tcf7l1**	Transcription factor 7 like 1	Unk	Unk	Aka Tcf3- TF involved in Wnt signal transduction
**Col4a1**	Collagen type 4 alpha 1	Unk	Unk	Collagen; in all basement membranes

Unk: Unknown.

## Discussion

We have identified surprising overlap between genes expressed in HSCs and genes that are expressed in the brain and encode proteins in protein interaction networks for neurological diseases such as ataxia and autism. We have shown that this relationship is not recapitulated by genes expressed in differentiated hematopoietic cells, but is specific to genes expressed in HSCs. Thus, these data reveal a previously underappreciated functional relationship and raise the possibility that additional genes critical for normal brain function might be candidates for regulating HSCs, and vice versa.

This finding is surprising given the generally low overlap between genes annotated to neuronal and hematologic phenotypes (Figure 6C). The fact that there is little correlation between mice with a nervous system phenotype and those with a reported hematopoietic stem cell phenotype as described in MGI, most likely reflects the limited set of phenotypes most investigators consider when studying their genes of interest. Our finding that Atxn1L, a gene identified from the Ataxia interactome, has a hematopoietic phenotype when ablated supports this concept. These data argue that this approach of highly focused phenotyping may obscure unexpected correlations that may have functional relevance. Cross-system analyses, particularly when functions might be predicted from computational approaches derived by mining available biological and *in silico* data, may be of significant value.

Our data clearly establish Atxn1L as a negative regulator of HSC function. By employing multiple functional assays we show that *Atxn1L^−/−^* HSCs are super-HSCs. They regenerate the blood of recipient mice to higher levels than WT HSCs, they recover more quickly from myeloablative treatment, and they exhibit better engraftment even after secondary transplantation, a rigorous measure of HSC self-renewal capacity. Although, there are now a number of genes that when ablated result in decreased stem cell function, there are relatively few that result in enhanced HSC activity [22]. Genes that act normally to restrain HSC activity, resulting in higher performance after KO, include *Cbl* [26], *Slug* [27], *Cdkn2c* (p18) [28] and *Gli1* [29]. Importantly, none of these genes showed significant down-regulation in *Atxn1L^−/−^* HSCs, suggesting that the mechanism of enhanced stem cell function in the *Atxn1L^−/−^* mice is distinct.

Many of the genes that affect HSC function impact the proliferation rate of HSCs [22]. Paradoxically, higher proliferation of HSCs is usually linked to lower HSC activity. For example, HSCs from *Irgm* KO mice show excessive proliferation and poor engraftment properties, owing to hyper interferon signaling [30], [31], and *Gfi1* mutant HSCs are also hyperproliferative and similarly defective [2]. While not well understood, this link between high HSC proliferation and poor engraftment probably relates to differentiation-associated HSC proliferation that ultimately depletes the stem cell pool. Consistent with this, some mutants that decrease HSC proliferation, for example *Gli1*, augment HSC function [29]. On the other hand, increased HSC proliferation can also be associated with enhanced HSC function: KO of *Slug* or *Gfi1b*, both putative transcriptional repressors, results in improved HSC bone marrow engraftment activity along with slightly increased HSC proliferation [27], [32], similar to our observations in *Atxn1L^−/−^* mice. Again, while not fully understood, moderately higher proliferation may enable more rapid engraftment after transplantation (similar to accelerated recovery of blood counts after 5FU) that, if not excessive, may also preserve stem cell function. These findings underscore the critical balance that is maintained to optimize the competing roles of stem cells in self-renewal and differentiation.

The molecular mechanism of the *Atxn1L^−/−^* HSC phenotype is not easy to establish at this time as no major pathways were altered in the gene expression analysis to suggest particular avenues for further study. Atxn1 and Atxn1L have both been shown to interact with the transcriptional repressor Capicua (Cic), which mediates a number of their downstream effects. In the lung, loss of the Atxn1/Atxn1L destabilizes Cic complexes leading to de-repression of activators of matrix metalloproteinases that in turn contribute to the lung alveolarization defects [11]. We have not examined protein levels of Cic in HSCs, but we do detect high expression of *Cic* in HSCs [7], which leaves open the possibility of a role for Cic in the hematopoietic phenotype as well. Ultimately, better understanding of the mechanisms that lead to enhanced stem cell function could lead to strategies to expand HSCs for bone marrow transplantation which, despite much effort, has still not been achieved.

More broadly, our work suggests the existence of molecular networks that are utilized in both brain and hematopoietic stem cells, but not their differentiated counterparts. Whether these networks are also used in other adult stem cells, as was recently suggested for imprinted genes [17] is an open question. Our work also suggests a paradigm for using cross-tissue bioinformatic analyses to identify new key regulators in blood or brain. While other genes are anecdotally linked in both systems, we expect many others could be probed. With the advent of the large-scale mouse phenotyping efforts stimulated by the knock-out mouse consortia, these types of analyses offer a parsimonious use of resources to efficiently identify important phenotypes and cross-tissue phenotype comparisons.

It is interesting to consider why this apparent relationship exists. The fact that neither HSCs nor most brain cells actively divide does not seem to be the cause, as our quiescence signature genes do not show the same enrichment as the HSC fingerprint (Figure 6). We speculate that there is either a relationship rooted in ontology or evolution that has not been previously noted, or that there is some underlying functional origin. For example, HSCs have a close relationship with other cells in their niche- perhaps they utilize a “synapse” to communicate with other key bone marrow components. Along these lines, a link has previously been noted between some genes with an impact on endothelial cell function and those involved in brain function. For example, classical axon-guidance cues also help guide blood vessel formation [33]. HSCs and endothelial cells have a close relationship that originates in their development. HSCs arise from specialized endothelial cells [34], [35] and co-express a number of key genes such as *Runx1*, *Sca1*, and *Scl/Tal1*. Thus, it is possible that underlying relationship between HSCs and the brain is also linked to their commonalities with endothelial cells. Systematic analyses with endothelial-specific genes of the type we have performed here would be required to probe this possibility further.

It is also possible that our observations of common brain-HSC networks may hold for humans. Some well known genetic syndromes have been recognized to exhibit both neurologic and hematologic components. For example, Alpha-Thalassemia mental Retardation X-linked syndrome (ATRX) is named for its involvement in both alpha thalassemia and mental retardation (OMIM 301040). Similarly, Ataxia-Telangiectasia (OMIM 208900) and Nijmegen Breakage syndrome, (OMIM 251260) have both hematologic and neurological manifestations. Furthermore, Autism patients may have higher frequencies of infections [36], which could suggest shared genetic etiology. Further studies to explore this intriguing link between the neurologic and hematopoietic system defects are clearly warranted.

## Materials and Methods

### Mice

All mice were backcrossed to the C57Bl/6 background and were housed in a specific-pathogen-free animal facility, AALAC-accredited, at Baylor College of Medicine (Houston, TX).

### Hematopoietic analyses

For peripheral blood analysis, transplant recipient mice (n = 8/genotype) were bled at 4, 8,12 and 16 weeks post transplantation. Red blood cells were lysed and samples were stained with CD45.1-APC, CD45.2-FITC, CD4-pacific blue, CD8-pacific blue, B220-pacific blue, B220-PE-cy7, Mac1-PE-cy7 and Gr-1-PE-cy-7 antibodies (BD Pharmingen, eBiosciences). FacsARIA, LSRII and FACS-Scan flow cytometers were used for analysis and cell sorting. Hematopoietic committed progenitors were analyzed based on expression of cell surface markers that can be identified using flow cytometry as described [37]. For complete blood counts, peripheral blood was collected from the retro-orbital plexus into tubes containing potassium EDTA (Sarstedt, Nümbrecht, Germany) from 8–10 week old WT and *Atxn1L^−/−^* mice (n = 10 mice/genotype) and analyzed with a Hemavet analyzer (Drew Scientific, TX, USA). LT-HSCs defined in text and legends. ST-HSCs: Sca-1^+^, c-kit^+^, CD34^+^, Flt3^−^; MPP: Sca-1^+^, c-kit^+^, CD34^+^, Flt3^+^; CLPs: Lineage^−^, IL7rα^+^, Sca-1^+^, c-kit^+^; CMP: Lineage^−^, IL7rα^−^, Sca-1^−^, c-kit^+^, CD34^+^, CD16/32^−^; GMP: Lineage^−^, IL7rα^−^, Sca-1^−^, c-kit^+^, CD34^+^, CD16/32^+^; MEP: : Lineage^−^, IL7rα^−^, Sca-1^−^, c-kit^+^, CD34^−^, CD16/32^−^.

### Bone marrow transplantation

Competitive bone marrow transplantation assays were performed by intravenous injection of admixed CD45.2 donor whole bone marrow cells with CD45.1 competitor bone marrow. Recipient C57Bl/6 mice had been lethally irradiated with a split dose of 10.5 Gy, 3 hours apart. Sex- and age-matched C57Bl/6 mice were used as competitors for every experiment. Eight recipient mice were used in each experiment (n = 8 mice/genotype), and each experiment was repeated at least twice. The competitor cell dose was kept constant at 250,000 cells in all transplants.

For the limiting dilution assays to determine repopulating units, we used 1.0×10^3^, 3.0×10^4^ and 1.0×10^5^ WT or *Atxn1L^−/−^* whole bone marrow cells pooled from (n = 3/genotype) CD45.2 sex- and age-matched mice (for the number of recipient animals in each dilution group, see Figure 2), mixed with 250,000 CD45.1 cells. Positive engraftment was scored based on multilineage repopulation of higher than 0.1%. The percentage of non-responders was calculated using the L-Calc software (StemCell Technologies).

HSC transplants were carried out as described above, but instead of whole bone marrow donor cells, purified HSCs were transplanted. Unless specified otherwise, HSCs were isolated using the side population (SP) method for Hoechst dye efflux [38], followed by KSL (c-Kit^+^, Sca1^+^, lineage^−^) and CD150^+^ staining. Recipient mice (n = 8/genotype) received 20 sorted HSCs and 250,000 WT competitor cells. Staining and isolation of HSCs were carried out as previously described [37].

For the secondary transplants, primary recipient mice were sacrificed 16 weeks post transplant and CD45.2 donor-derived HSCs were isolated as described above. Fifty HSCs were transplanted into secondary CD45.1 recipients, along with 250,000 CD45.1 competitor whole bone marrow.

### Homing assay

Homing efficiency of donor cells into the recipient bone marrow was characterized in two ways. First, 30,000 KSL cells from pooled WT and *Atxn1L^−/−^* mice (CD45.2) were isolated and transplanted into lethally irradiated CD45.1 recipient mice (n = 5/genotype). Eighteen hours after the transplant, the recipient mice were sacrificed and their bone marrow was analyzed for the presence of CD45.2 positive cells using flow cytometry. A fraction of the whole bone marrow was also plated on methylcellulose medium in 32 mm dishes (n = 5 dishes/genotype). Controls included irradiated recipient mice that received no CD45.2 marrow. The resulting colonies are derived from the CD45.2 donor cells that homed into the recipient mice bone marrow. Thus, 12 days after plating, colonies were counted in each well to assess the homing efficiency of donor cells.

### Methylcellulose cultures

HSCs were identified using the Hoechst dye efflux method along with positive staining for c-kit, Sca-1, CD150 and excluding lineage positive cells. Single HSCs were sorted in 96-well plates containing methylcellulose medium (StemCell Technologies). The number of colonies was counted at days 4, 7 and 14 and scored based on morphology on day 10.

For the *in vitro* colony proliferation assay, HSCs were sorted and plated into 6-well plates containing methylcellulose, 100 HSCs per well. Seven days later, single colonies were picked and resuspended in HBSS medium contain FBS (Gibco). The cell suspension was washed twice, stained with PI in sodium citrate and analyzed by flow cytometry.

### Retroviral transduction

MSCV-Atxn1L-IRES-GFP and MSCV-IRES-GFP vectors were packaged using HEK293T cells by co-transfecting with pCL-Eco [39]. Mice were treated with 5-fluorouracil (150 mg/Kg body weight, American Pharmaceutical Partners) 6 days before harvesting the whole bone marrow. The bone marrow was enriched for Sca-1 expressing cells using magnetic selection (AutoMACS, Miltenyi), transduced with the retrovirus as previously described [7], and grown in culture. After 48 hours, cells were collected, stained for Sca-1, c-kit and lineage markers, and GFP+ KSL cells were sorted and plated into 6-well plates containing methylcellulose medium. Ten days later, the number of colonies in each well was counted.

### Proliferation assays

Whole bone marrow from WT and *Atxn1L^−/−^* mice (n = 5/genotype) was isolated and stained for different hematopoietic progenitor populations. Cells were then fixed and stained for either BrdU or Ki-67 according to the BrdU staining protocol supplied by the manufacturer (BD-Pharmingen).

### 5-fluorouracil experiments

To determine how WT and *Atxn1L^−/−^* mice respond to stress, mice were treated with the chemotherapeutic drug, 5-FU. To determine the survival rate, mice (n = 5/genotype) received one injection of 5-FU (150 mg/Kg body weight) and analyzed for proliferation or apoptosis 3, 5 and 7 days later by flow cytometry.

In order to assess hematopoietic recovery after stress, WT and *Atxn1L^−/−^* mice (n = 10/genotype) were treated once with 5-FU and their peripheral blood counts were monitored every three days for 28 days using the Hemavet analyzer (Drew Scientific, TX, USA).

### Annexin-V assays

Annexin-V staining was used to assess cell death and apoptosis. Briefly, cells were harvested and stained with the markers of interest according to the staining protocol described above. Cells were washed twice with cold PBS and incubated at room temperature in 1× binding buffer (10 mM HEPES, 140 mM NaCl, 2.5 mM CaCl2) containing Annexin V-APC (BD-Pharmingen). Cells were analyzed by flow cytometry within one hour of staining.

### Microarray analysis

HSCs were purified as described above. Cells were purified from 8-week-old mice. Approximately 30,000 HSCs from WT and *Atxn1L^−/−^* mice were purified for RNA isolation. RNA was isolated using the RNAqueous kit (Ambion, Austin, TX, USA), and treated with DNase I. The RNA was linearly amplified using two rounds of T-7 based *in vitro* transcription using the MessageAmp kit (Ambion). The RNA was subsequently labeled with biotin-conjugated UTP and CTP (Enzo Biotech). The amplified RNA was hybridized to MOE430.2 chips according to standard protocol at the BCM Microarray core (Houston, TX). Data were analyzed by GCRMA with correction for false-discovery [40]. Data can be found in GEO, with accession number: GSE44285.

### Bioinformatics analysis

In all cases of overlap analysis between gene sets, a Fisher's exact test was performed to determine p-values and statistical significance. We also generated Z-scores to measure the deviation between the observed overlap (number of genes in common between two sets) and what would be expected if one set were fixed and a random set was generated to overlap with it (eg. array overlap with P-sig, Q-sig and the HSC fingerprint). The expected overlap size was determined as the product between frequency of the fixed set and the size of the comparator set. The frequency was determined as the number of genes in the fixed set divided by the number of all genes that could be sampled; the size of the comparator set was limited to the size of the comparator overlapped with the sample universe (i.e. when doing cross platform comparison, the comparator size was limited to the subset of the comparator represented among the universe of the fixed set. To generate the network in Figure 6D, we used homologene to map mouse gene symbols to human orthologs. We then identified all interactions in the interactome where one of the partners was expressed in HSC according to the HSC fingerprint dataset [7]. We used Cytoscape to generate the network image. We identified the gene products that were additionally identified as being HSC fingerprint genes by coloring them red. HSC expressed genes are colored pink.

For microarray data we used the R Bioconductor package GCRMA to process the low-level intensity data. We used the limma package to generate T-statistics and moderated p-values. We used the Bioconductor package mouse4302 v2.2 to determine the gene symbols for the probe sets on the array, and we used the same release to compare both the previously published HSC gene lists and our new array results.

### Ethics statement

All animal work has been conducted according to national and international guidelines. The institutional animal care and use committee (IACUC) at Baylor College of Medicine approved the animal protocols for the work described herein. No human or primate samples were used for this work (data mining only).

## Supporting Information

Table S1 Data for Figure 6A and 6C.
(XLSX)

Table S2 A compendium of genes shown to affect HSC function was analyzed for neurological phenotype. Genes with a reported HSC phenotype were also likely to have a neurological phenotype (Z = 4.06; p = 5.72×10^−5^).
(XLSX)

Table S3 HSC-specific genes in the Ataxia interactome.
(DOCX)

Table S4 HSC-specific genes in the autism interactome.
(DOCX)
