# Supplementary material for: Ataxin1L Is a Regulator of HSC Function Highlighting the Utility of Cross-Tissue Comparisons for Gene Discovery
Source: PLoS Genet. 2013 Mar 28;9(3):e1003359. doi: 10.1371/journal.pgen.1003359 (PMC3610904; doi:10.1371/journal.pgen.1003359)
Supplement: Table S3 — HSC-specific genes in the Ataxia interactome. (DOCX) [file pgen.1003359.s003.docx]

Table S3 . HSC-specific genes in the Ataxia interactome

| Affy probe ID | Symbol | OverallBrain | Amygdala | Cerebellum | Hypothalamus | HSC mean | Non-HSC mean |
| --- | --- | --- | --- | --- | --- | --- | --- |
| 1415923_at | **Ndn** | 9.49 | 9.17 | 9.42 | 9.86 | 7.40 | 3.32 |
| 1416783_at | **Tac1** | 8.02 | 9.23 | 4.69 | 9.04 | 5.73 | 3.67 |
| 1418049_at | **Ltbp3** | 7.68 | 6.76 | 8.91 | 7.47 | 8.21 | 3.18 |
| 1424051_at | **Col4a2** | 7.00 | 6.62 | 7.35 | 7.22 | 8.12 | 2.86 |
| 1424800_at | **Enah** | 8.50 | 8.00 | 8.55 | 8.77 | 5.64 | 2.79 |
| 1425458_a_at | **Grb10** | 7.94 | 7.11 | 7.66 | 8.71 | 9.90 | 3.50 |
| 1425924_at | **Mdfi** | 5.58 | 4.91 | 6.98 | 5.17 | 6.32 | 3.35 |
| 1426431_at | **Jag2** | 7.62 | 7.25 | 7.74 | 8.01 | 6.98 | 3.09 |
| 1428816_a_at | **Gata2** | 6.00 | 5.23 | 6.60 | 5.88 | 5.23 | 3.43 |
| 1433924_at | **Peg3** | 10.24 | 9.48 | 10.46 | 10.48 | 6.50 | 2.94 |
| 1434033_at | **Tle1** | 7.78 | 7.37 | 7.98 | 8.03 | 5.97 | 3.77 |
| 1437833_at | **Ltbp3** | 7.68 | 6.76 | 8.91 | 7.47 | 10.35 | 3.85 |
| 1439527_at | **Pgr** | 6.68 | 6.46 | 6.59 | 6.80 | 6.11 | 2.69 |
| 1441573_at | **Scmh1** | 7.72 | 7.19 | 8.75 | 7.72 | 5.13 | 3.10 |
| 1448169_at | **Krt18** | 5.16 | 4.77 | 5.90 | 5.15 | 10.38 | 3.67 |
| 1450062_a_at | **Maged1** | 10.07 | 9.79 | 10.05 | 10.29 | 7.79 | 3.25 |
| 1450117_at | **Tcf7l1** | 7.30 | 6.63 | 8.47 | 7.19 | 5.75 | 3.17 |
| 1450333_a_at | **Gata2** | 6.00 | 5.23 | 6.60 | 5.88 | 9.28 | 3.06 |
| 1452035_at | **Col4a1** | 6.54 | 6.17 | 7.01 | 6.53 | 8.00 | 2.92 |
| 1455792_x_at | **Ndn** | 9.49 | 9.17 | 9.42 | 9.86 | 12.19 | 3.69 |
| 1456189_x_at | **Ltbp3** | 7.68 | 6.76 | 8.91 | 7.47 | 7.59 | 3.47 |
| 1416778_at | **Sdpr** | 5.01 | 4.60 | 5.71 | 4.54 | 7.85 | 3.38 |
| 1416945_at | **Ptov1** | 8.82 | 8.41 | 9.56 | 8.75 | 5.40 | 2.54 |
| 1417336_a_at | **Sytl4** | 4.86 | 4.14 | 5.17 | 5.81 | 8.29 | 3.35 |
| 1417872_at | **Fhl1** | 8.81 | 8.29 | 9.42 | 8.82 | 10.60 | 2.98 |
| 1420647_a_at | **Krt8** | 5.68 | 5.36 | 6.15 | 5.74 | 6.54 | 2.62 |
| 1421461_at | **Mpl** | 5.98 | 5.40 | 6.67 | 5.92 | 11.10 | 3.57 |
| 1421841_at | **Fgfr3** | 9.26 | 8.62 | 9.43 | 9.42 | 7.88 | 3.14 |
| 1423691_x_at | **Krt8** | 5.68 | 5.36 | 6.15 | 5.74 | 6.51 | 2.62 |
| 1424410_at | **Ttc8** | 7.02 | 6.58 | 7.58 | 7.07 | 6.40 | 3.20 |
| 1428680_at | **Cds1** | 8.48 | 7.66 | 9.27 | 8.29 | 5.52 | 3.39 |
| 1430043_at | **Ttc19** | 8.69 | 8.27 | 9.10 | 9.04 | 8.07 | 2.86 |
| 1430352_at | **Adamts9** | 5.49 | 5.29 | 5.73 | 5.33 | 5.25 | 3.12 |
| 1431900_a_at | **Foxa3** | 5.23 | 4.61 | 5.88 | 5.25 | 6.93 | 2.98 |
| 1435989_x_at | **Krt8** | 5.68 | 5.36 | 6.15 | 5.74 | 6.38 | 2.61 |
| 1436244_a_at | **Tle2** | 7.18 | 6.04 | 9.32 | 6.73 | 8.00 | 3.10 |
| 1438325_at | **Mecom** | 4.77 | 3.98 | 5.38 | 4.49 | 12.56 | 3.47 |
| 1439368_a_at | **Slc9a3r2** | 8.14 | 7.21 | 9.20 | 7.86 | 6.38 | 3.48 |
| 1440104_at | **Ranbp2** | 8.55 | 8.08 | 9.40 | 8.37 | 7.05 | 3.55 |
| 1441072_at | **Celf2** | 8.66 | 9.32 | 9.12 | 8.82 | 7.24 | 2.86 |
| 1443838_x_at | **Fads2** | 10.46 | 10.18 | 10.35 | 10.69 | 5.31 | 2.78 |
| 1444507_at | **Usp53** | 8.08 | 7.17 | 8.77 | 7.85 | 5.63 | 2.99 |
| 1444620_at | **Tcf12** | 8.40 | 7.54 | 9.58 | 8.04 | 5.67 | 3.32 |
| 1447863_s_at | **Nr4a2** | 7.63 | 7.57 | 8.76 | 8.01 | 9.15 | 3.60 |
| 1448665_at | **Dmd** | 7.31 | 6.97 | 7.73 | 7.41 | 7.92 | 3.61 |
| 1448754_at | **Rbp1** | 7.63 | 7.29 | 8.23 | 7.30 | 6.48 | 2.71 |
| 1449431_at | **Trpc6** | 5.89 | 6.08 | 6.28 | 5.96 | 9.12 | 4.31 |
| 1452220_at | **Dock1** | 6.53 | 5.85 | 7.03 | 6.28 | 5.70 | 3.35 |
| 1454243_at | **Ick** | 7.07 | 6.27 | 7.63 | 7.19 | 5.29 | 2.59 |

Shown are the expression values from microarray gene expression profiling of the genes that are HSC-specific (fingerprint genes) and are found in the ataxia interactome. Note that expression values are not directly comparable between the tissues, as the brain data are from human exon arrays, and the HSC data are from murine gene expression arrays.
